# Association of the independent polymorphisms in CDKN2A with susceptibility of acute lymphoblastic leukemia

**DOI:** 10.1042/BSR20180331

**Published:** 2018-06-27

**Authors:** Xueyan Zhou, Fei Liao, Junlong Zhang, Yun Qin, Heng Xu, Zhenyu Ding, Yan Zhang, Feng Zhang

**Affiliations:** 1 Department of Thoracic Oncology, Cancer Center, West China Hospital, West China Medical School, Sichuan University, Chengdu, Sichuan 610041, China; 2 Department of Laboratory Medicine, Precision Medicine Center, West China Hospital, Sichuan University, Chengdu, Sichuan 610041, China; 3 Department of Radiology, West China Hospital, Sichuan University, Chengdu, Sichuan 610041, China; 4 Department of Clinical Laboratory, Core Facility, Quzhou People’s Hospital, Quzhou, Zhejiang 324000, China

**Keywords:** ALL, CDKN2A, rs3731217, rs3731249, susceptibility

## Abstract

Acute lymphoblastic leukemia (ALL) is the most common cancer in children, and alterations in *CDKN2A* were considered to play an important role on leukemogenesis. Two single nucleotide polymorphisms (SNPs) at *CDKN2A* locus were identified to impact on ALL susceptibility via genome wide association studies, and followed by multiple subsequent replication studies at the specific hits. Here, we conducted a systematic review and meta-analysis to re-evaluate the association of both SNPs (rs3731217 and rs3731249) with ALL susceptibility by gathering the data from 24 independent studies, totally containing 7922 cases/21503 controls for rs3731217 and 6295 cases/24191 controls for rs3731249. Both SNPs were significantly associated with ALL risk (odds ratio [OR] = 0.72 and 2.26 respectively), however, exhibit race-specific pattern. In summary, our meta-analysis indicated that two SNPs at *CDKN2A* locus are associated with ALL susceptibility independently mainly in Caucasians. Future large-scale studies are required to validate the associations in other ethnicities.

## Introduction

Acute lymphoblastic leukemia (ALL) is the most common pediatric cancer worldwide [1]. Genetic susceptibility basis of such deadly malignancy has been largely investigated, identifying somatically acquired genomic aberrations, which is hallmark of the majority ALL cases [2, 3]. For instance, focal or whole-arm deletion of *CDKN2A* gene, which encodes two important tumor proteins (p14^ARF^ and p16^INK4A^), is commonly observed in leukocytes of ALL patients [1, 4]. Recently, inherited predispositions to ALL susceptibility have also been recognized by conducting genome-wide association studies (GWASs) across diverse ethnicities, identifying common variants at several genetic loci, including single nucleotide polymorphisms (SNPs) in *ARID5B, IKZF1, CEBPE, CDKN2A, PIP4K2A-BMI1*, and *GATA3* [5–9]. However, variations even inconsistence were observed in the replication studies, possibly because of the diverse clinical characteristics, including ethnicity, age and subtypes [10–13]. Besides the top SNPs identified in intronic or intergenic regions, exome-array based GWAS was also conducted to systematically investigate the relationship between coding variants and ALL susceptibility in Caucasian population with 1773 cases and 10,448 controls [14]. Only one common missense variant at *CDKN2A* (rs3731249) reaches genome-wide significance. By using imputation or array-based approaches, association of this SNP with ALL susceptibility has been validated among multiethnic populations in another three independent studies [15–17]. Further investigation on the interaction between germline variants and somatic alterations, we and other group found that patients with heterozygous genotype of rs3731249 were tend to loss the expression of wild-type *CDKN2A* in their leukemia cells, either through loss of heterozygosity or allele-specific post-transcriptional inactivation [14]. Moreover, we conducted *in vitro* experiments to further test the influence of variants at rs3731249 on leukemogenesis, and found p14^ARF^ encoded by *CDKN2A* gene with variant allele of rs3731249 loses its ability to suppress leukemic transformation compared with the wild-type p14^ARF^, indicating rs3731249 is a potential causal variant to ALL susceptibility [14]. Therefore, two SNPs in *CDKN2A* were identified as ALL-associated GWAS signals, highlighting the importance of *CDKN2A* in leukemogenesis in both germline and somatic levels.

In the present study, we incorporated all the relevant publications, and collected the information from 24 independent studies [9, 14–29], 7922 cases/21503 controls for rs3731217 and 6295 cases/24181 controls for rs3731249 were gathered. Finally, meta-analysis was conducted with a large sample size with diverse ethnicities to investigate the effects of *CDKN2A* SNPs (including rs3731217 and rs3731249) on ALL risk.

## Methods

### Literature and study acquisition

We systematically retrieved all the related papers in PubMed (http://www.ncbi.nlm.nih.gov/pubmed) and Google Scholar (http://scholar.google.com/) date to November 5, 2015 utilizing the following retrieve phrases: “rs3731217” or “rs3731249” or “acute lymphoblastic leukemia” and “polymorphisms” and “CDKN2A” or “childhood acute lymphoblastic leukemia” and “susceptibility” or “genetic polymorphism” and “acute lymphoblastic leukemia” and “GWAS”, or “germline variants” and “acute lymphoblastic leukemia”. All papers (*N*=131) were confined to English. After that, initial screening of the titles along with the abstracts was executed by two independent reviewers, overlapped studies as well as papers that do not meet our motif were discarded (*N*=87). Following, full-text based filtering was conducted among the left studies and reserved eligible papers according to the criteria listed below: (1) studies adopted case–control design; (2) assessed the effect of rs3731217 or rs3731249 polymorphisms on ALL risk; (3) offering the sample size; (4) giving the genotype counts or detail information to infer the genotypes; and (5) data from each study without overlap. When repeated data encountered, only the latest publication or most detailed data were included.

### Data extraction and verification

Two independent reviewers extracted information from each study based on the following contents: first author, publication date, country, ethnicity, study design, sample size, genotyping platform, and genotypes. When datasets were not accessible or partial for the requisite data, corresponding authors were connected for additional information. Gathering information from the included studies was listed in Tables 1 and 2.

**Table 1:** Principle characteristics of the studies included in the meta-analysis for rs3731217 polymorophism at CDKN2A locus

Author	Year	Country or Institution	Ethnicity	Study design	No. of cases	No. of control	Genotyping platform	Kind of genotypes
Sherborne et al.	2010	U.K.	Caucasian	GWAS	504	1438	Illumina array	TT/TC/CC
Vijayakrishnan et al.	2010	U.K.	Asian	Replication	190	182	KASP	TT/TC/CC
Orsi et al.	2012	France	Caucasian	GWAS	441	1984	Illumina array	TT/TC/CC
Burmeister et al.	2014	Berlin	Caucasian	Replication	322	1516	TaqMan	TT/TC/CC
Peyrouze et al.	2012	France	Caucasian	Replication	150	180	TaqMan	T/G
Pastorczak et al.	2011	Poland	Caucasian	Replication	398	731	KASP	TT/TC/CC
Walsh et al.	2015	U.S.A.	Hispanic	Replication	321	454	Illumina array	T/G
			Caucasian	Replication	980	2624	Affymetrix 6.0	T/G
			African-American and Hispanic	Replication	163	201	TaqMan	T/G
Vijayakrishnan et al.	2015	U.K.	Caucasian	GWAS	824	5200	Illumina array	TT/TC/CC
			Caucasian	GWAS	834	2024	Illumina array	TT/TC/CC
Chokkalingam et al.	2013	U.S.A.	Hispanic	Replication	300	406	Sequenom iPlex	T/G
			Caucasian	Replication	225	369	Sequenom iPlex	T/G
Hungate et al.	2016	U.S.A.	Caucasian	Replication	1406	1399	Array based Imputation	TT/TC/CC
			African-American	Replication	203	1363	Array based Imputation	TT/TC/CC
			Hispanic	Replication	391	1008	Array based Imputation	TT/TC/CC
Kreile et al.	2016	Latvia	Caucasian	Replication	76	121	PCR-RFLP	T/C
Gharbi et al.	2016	Tunis	Afrian	Replication	58	150	PCR	TT/TC/CC
Al-absi et al	2017	Yemen	Asian	Replication	136	153	Fluidigm 192.24 Dynamic Array	TT/TC/CC

Abbreviations: GWAS, genome-wide association study; KASP, kompetitive allele specific PCR; PCR, polymerase chain reaction; PCR-RFLP, polymerase chain reaction-restriction fragment length polymorphism.

**Table 2:** Principle characteristics of the studies included in the meta-analysis for rs3731249 polymorophism at CDKN2A locus

Author	Year	Country or Institution	Ethnicity	Study design	No. of cases	No. of controls	Genotyping platform	Kind of genotyoes
Heng et al.	2015	U.S.A.	Caucasian	GWAS	1773	10448	Illumina array	TT/CT/CC
			Caucasian	Replication	410	1599	Illumina array	TT/CT/CC
Healy et al.	2007	U.S.A.	Caucasian	Replication	240	277	PCR	TT/CT/CC
Vijayakrishnan et al.	2015	U.K.	Caucasian	GWAS	824	5200	Illumina array	TT/CT/CC
			Caucasian	GWAS	834	2024	Illumina array	TT/CT/CC
			Caucasian	Replication	519	1016	KASP	TT/CT/CC
Walsh et al.	2015	U.S.A.	Hispanic	Replication	321	454	Illumina array	T/C
			Caucasian	Replication	980	2624	Affymetrix array	T/C
			African-American and Hispanic	Replication	163	201	TaqMan	T/C
Gutierrez-Camino et al.	2017	Spain	Caucasian	Replication	231	338	PCR	TT/CT/CC

Abbreviations: GWAS, genome wide association study; KASP, Kompetitive Allele Specific PCR; PCR, polymerase chain reaction.

### Choice of genetic model

The rs3731249 polymorphism owns variant T allele and wild-type C allele. We plan to investigate the relationship between rs3731249 polymorphism and childhood acute lymphoblastic leukemia risk by utilizing the allele model (T allele vs. C allele), the dominant model (TT + TC vs. CC), and the recessive model (TT vs. TC + CC). Identically, the rs3731217 polymorphism contains wild-type allele T and variant allele C. We also employed the similar genetic model to inquiry the association between rs3731217 polymorphism and childhood acute lymphoblastic leukemia predisposition.

### Heterogeneity test

Heterogeneity among studies was assessed through *Q* statistic and *I*^2^ statistic [30], where *Q* approximately obeys a *χ*^2^ distribution with *k* − 1 degrees of freedom, while *k* is the number of studies. Specially, *P* value can be employed to test the significance level of heterogeneity; *I*^2^ = [*Q − (k −* 1)]/*Q*100%, varying from 0 to 100%. Generally, *I*^2^ = 50% is a threshold value, when *I*^2^ < 50% and *P* value >0.1, heterogeneity among studies were acceptable and fixed-effect model is more suitable to compute the merge OR and 95% CI. On the contrary, if *I*^2^ > 50% and *P* value <0.1, indicating highly heterogeneity, were existed in those studies and random-effect model was utilized to compute the merge OR and 95% CI. When necessary, subgroup analyses can be employed.

### Sensitivity analysis

Sensitivity test carried out by omitting each of the studies discussed the association of rs3731217 (or rs3731249) with ALL susceptibility, pooled OR and 95% CI are not of significant difference (Supplementary Tables S5 and S6), which in turn certified the robustness of the relationship between rs3731217 or rs3731249 and ALL predisposition.

### Paper quality assessment

The quality estimation of the researches was based on the methodological quality assessment scale, which was adjusted from the Newcastle–Ottawa assessment scale for case–control studies (http://www.ohri.ca/programs/clinical_epidemiology/oxford.asp). The scales evaluated study qualities in several aspects (Supplementary Table S10): (1) inclusion and exclusion criteria of patients, (2) source of controls, (3) comparability of cases and controls, (4) sample size, (5) quality control of genotyping methods, and (6) HWE. Strictly obey the principles, two investigators separately evaluated and scored each study and the final results reached an agreement with a third author participated in the discussion. The final score showed in Supplementary Tables S11 and S12, and the higher the score, the better is the quality of the study.

### Publication bias analysis and Hardy–Weinberg equilibrium (HWE) test

According to the Cochrane Handbook for Systematic Reviews for Interventions, we examined the potential publication bias for all the rs3731217 studies (or rs3731249) by Begg’s funnel plot and Egger’s test (Supplementary Figures S1 and S2). Further, HWE was assessed by Chi-square test among the included studies in both SNPs studies respectively (Supplementary Tables S8 and S9).

## Results

### Study characteristics

**Figure 1:**
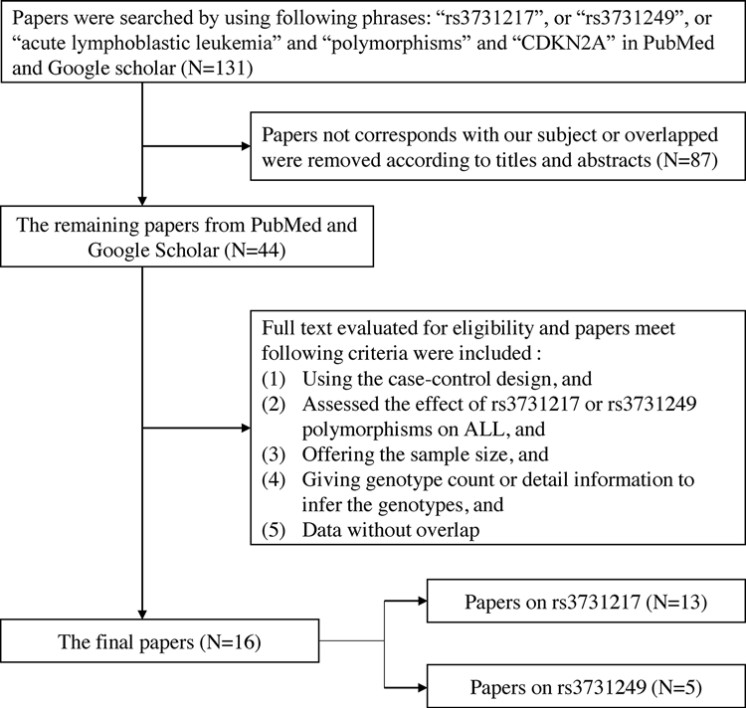
Flow chart of included studies for analyzing the association between rs3731217 and rs3731249 polymorphisms with ALL susceptibility Abbreviation: ALL, acute lymphoblastic leukemia.

Through literature search with keywords (see Methods), 24 independent researches manifested in 16 literatures met the inclusion criteria, and were selected for meta-analyses (Figure 1), describing association between ALL susceptibility and the SNPs at *CDKN2A* locus. For rs3731217, association of this SNP with ALL susceptibility was first reported in 2010 through GWAS approach, and investigated in other three GWASs or follow-up candidate studies. For the missense SNP rs3731249 (A148T), its influence on ALL risk was first reported in 2015, and was validated in another three studies. Since rs3731249 and rs36228834 are in perfect linage disequilibrium (LD) with each other (*r*^2^ = 1) in Caucasians, an earlier study can thus be included in the meta-analysis for rs3731249. The characteristics of 19 studies on rs3731217 and 10 studies on rs3731249 were summarized in Tables 1 and 2 respectively.

### Meta-analysis of the rs3731217 polymorphism and ALL susceptibility

**Figure 2:**
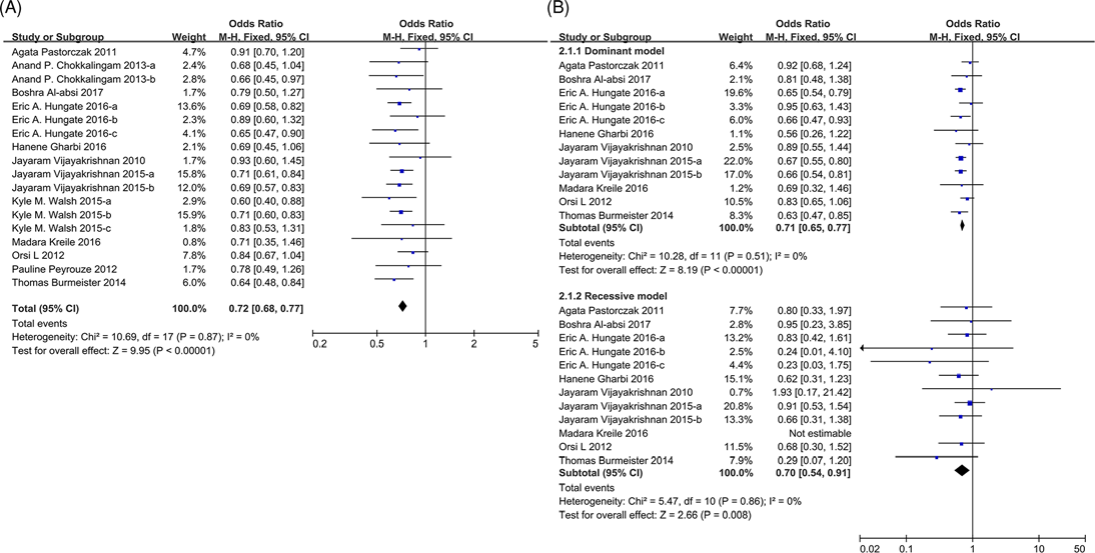
Forest plots of ALL predisposition associated with rs3731217 polymorphism under genetic models (**A**) Allelic model analysis (C vs. T) of rs3731217 and ALL risk. (**B**) Dominant (TC + CC vs. TT) and recessive (CC vs. TC + TT) model analysis of rs3731217 and ALL risk. Studies were plotted refer to the first author followed by publication year. For each research, the estimates of OR and its 95% CI are exhibited with square and a horizontal line. The area of the squares reflects the weight. The diamond represents the summary OR and 95% CI. Abbreviations: ALL, acute lymphoblastic leukemia; CI, confidence interval; OR, odds ratio.

**Figure 3:**
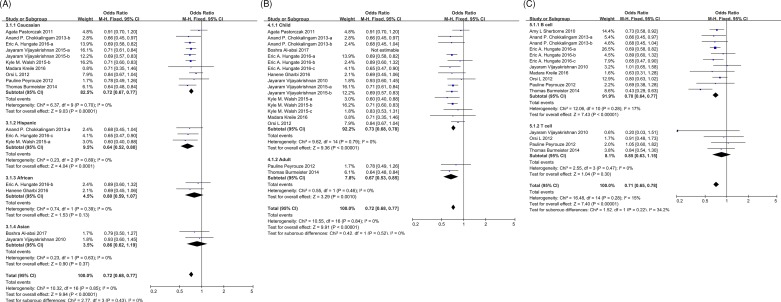
Forest plots of ALL suceptibility associated with rs3731217 polymorphism across ethnicity, age, and immunophenotype subtypes under the allelic model Forest plots of ALL susceptibility associated with rs3731217 polymorphism across ethnicity (**A**), age (**B**), and immunophenotype (**C**) subtypes under the allelic model (C vs. T). For each study, the estimates of OR and its 95% CI are plotted with square and a horizontal line. The area of the squares reflects the weight. The diamond represents the summary OR and 95% CI; Abbreviations: ALL, acute lymphoblastic leukemia; CI, confidence interval; OR, odds ratio.

Ninteen studies assessed the association between rs3731217 and ALL susceptibility with a total of 7922 cases and 21503 controls. As no significant heterogeneity was observed in the allele model (*P*=0.87 and *I*^2^ = 0%, Figure 2A), we applied fixed-effect model to conduct the meta-analysis, and found that C allele significantly exhibited a 0.72-fold (odds ratio [OR] = 0.72, 95% confidence interval [CI]: 0.68–0.77) increased risk to develop ALL (*P*<0.00001, Figure 2A and Supplementary Table S2) compared with T allele. In addition, no heterogeneity was observed in the dominate model (*P*=0.51 and *I*^2^ = 0%) and recessive model (*P*=0.86 and *I*^2^ = 0%) among 12 out of 19 studies that individual genotypes can be got (Figure 2B and Supplementary Table S1). Not surprisingly, results consistently exhibited that both genotypes CC and CT could lower the ALL predisposition. Next, we examined the effect of rs3731217 across ethnicities, age and ALL subtypes, no association was observed in Asian or T-linage ALL patients (Figure 3A–C), possibly because of the small sample size for patients with these clinical characteristics in the selected reports.

### Meta-analysis of the rs3731249 polymorphism and ALL susceptibility

**Figure 4:**
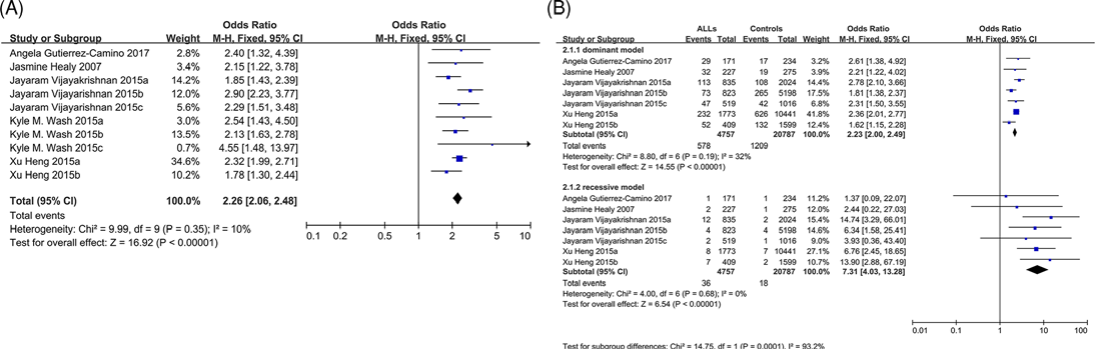
Forest plots of ALL predisposition associated with rs3731249 polymorphism under genetic models (**A**) Allelic model analysis (T vs. C) of rs3731249 and ALL risk. (**B**) Dominant (TC + TT vs. CC) and recessive (TT vs. TC + CC) model analysis of rs3731249 and ALL risk. Studies were plotted refer to the first author followed by publication year. For each research, the estimates of OR and its 95% CI are exhibited with square and a horizontal line. The area of the squares reflects the weight. The diamond represents the summary OR and 95% CI. Abbreviations: ALL, acute lymphoblastic leukemia; CI, confidence interval; OR, odds ratio.

Five articles assessed the association between rs3731249 and ALL susceptibility with a total of 6295 cases and 24181 controls. As rs36228834 is in prefect LD with rs3731249 in Caucasians, another study was also included to conduct this meta-analysis. Since no significant heterogeneity was observed in the allele model (*P*=0.35 and *I*^2^ = 10%, Figure 4A and Supplementary Table S4), we used fixed model to estimate the influence of the variant allele, and found that the minor allele (T) was significantly augment the ALL risk (*P*<0.00001, OR = 2.26, and 95% CI: 2.06–2.48). In addition, no heterogeneity was observed in the dominate model (*P*=0.19 and *I*^2^ = 32%) and recessive model (*P*=0.68 and *I*^2^ = 0%) among 7 out of 10 studies that individual genotypes can be got (Figure 4B and Supplementary Table S3). Given rs3731249 was located in the coding region of an important tumor suppressor gene CDKN2A, and induced alanine to threonine alteration in 148 position (A148T), allelic imbalanced was evaluated in somatic leukemia cells from individuals with heterozygous genotype of rs3731249 in 15 and 35 cases in two independent studies and indicated the variant allele preferentially retained significantly through either copy number variation or post-transcriptional inactivation during leukemogenesis.

### Publication bias analysis and sensitivity analysis

We utilized Begg’s test and Egger’s test to measure the publication bias for the all models for both SNPs, no evidence of obvious asymmetry was observed (Supplementary Figures S1 and S2). The result of sensitivity analysis showed that the association between rs3731217 (or rs3731249) and ALL risk doesn’t significantly fluctuate when removing each of the studies (Supplementary Tables S5 and S6).

### Ethnic diversity and LD pattern of SNPs at CDKN2A locus

Variant allele of rs3731217 was common across all ethnicities, while variant allele of rs371249 was only common in Caucasians and Hispanics, rare in Africans, even not observed in Asians. It could be the reason that no association between rs3731217 with ALL risk if rs3731249 was tagged by this SNP, we next investigated the genetic characteristic of CDKN2A locus across ethnicities, and identified the similar LD pattern between Caucasians and Hispanics, much less extensive in Africans and Asians. However, rs3731217 and rs3731249 were not in the same LD region, with *r*^2^<0.01 in Caucasians and Hispanics based on 1000 genome data (Supplementary Figure S3), which was consistent with the fact that these two SNPs associated with ALL risk independently in conditional model.

### Causal variant candidate determination

**Figure 5:**
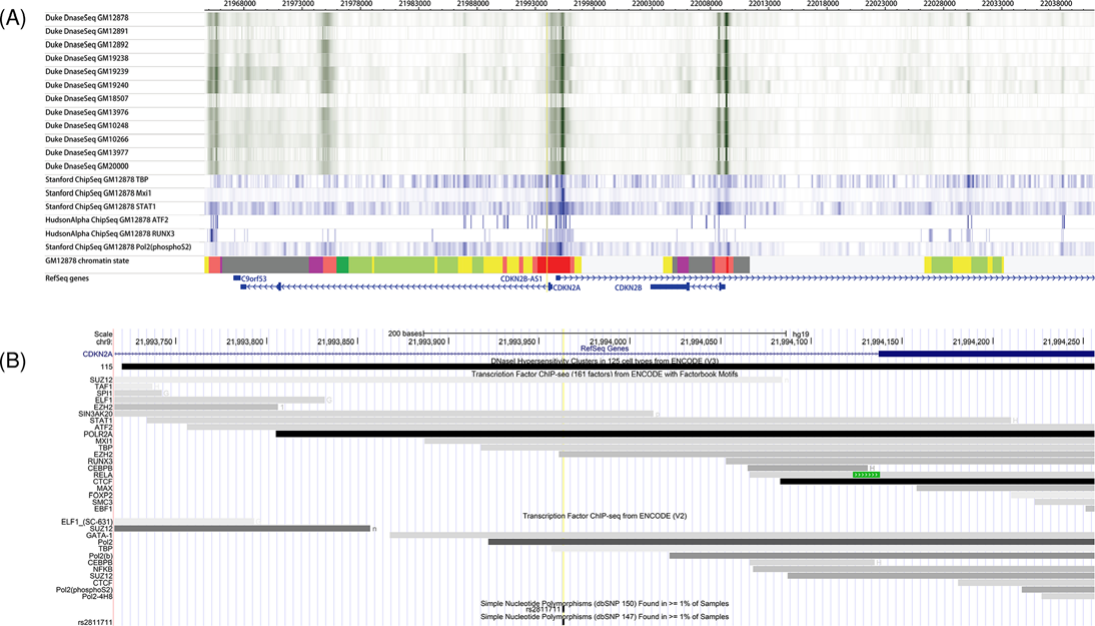
Epigenomic regulation signals at CDKN2A locus (**A**) Dnase-sequencing and Chiq-seq signals in the overlapped LD block (described in Supplementary Table S7) at Chr9:21956078-22039426 in Caucasians, the yellow line indicates the location of rs2811711 and information acquired from the WashU Epigenome Browser (http://epigenomegateway.wustl.edu/). (**B**) DNA- binding proteins around the rs2811711 locus obtained from the UCSC Genome Browser (http://genome.ucsc.edu/) and the yellow line indicates the location of rs2811711. Chromosomal locations are based on hg19; LD, linkage disequilibrium.

Variant allele of rs3731249 induced amino acid change in p16^INK4A^, protein product of *CDKN2A*, and reduced the tumor suppression effect of p16^INK4A^, suggesting rs3731249 act as a causal variant for ALL susceptibility. We next investigated the potential causal variant tagged by rs3731217 by using webtool (Haploreg), because association of this SNP with ALL can’t explained by rs3731249. Totally 17 known SNPs showed moderate LD (*r*^2^ > 0.4) with rs3731217 in Caucasians (Chr9:21956078-22039426) (Supplementary Table S7). Among these SNPs, rs2811711 is located within the region marked by activated promoter or enhancer histones in multiple tissue types, especially in B cells, based on the chromatin state information from Roadmap and ENCODE database (Figure 5A), and DNA-binding proteins (i.e. POL2, TBP, and GATA1) can also bind the rs2811711-located spot strongly (Figure 5B), suggesting the potential important role of rs2811711 on CDKN2A regulation (e.g. gene expression level). Moreover, variant allele frequency of rs2811711 is high in Caucasians, but no detected in Asians, which could explain the different association status of rs3731217 in diverse ethnic populations. Although further functional experiments are needed, rs2811711 could be considered as a causal variant candidate for ALL susceptibility.

## Discussion

A series of GWAS approaches had identified at least several loci those are significantly associated with ALL susceptibility. After the extensive replication studies, some loci can be validated in all independent multiethnic cohorts (e.g. *ARID5B*), whereas inconsistent associations were noticed in others (e.g. *CDKN2A*). Considering CDKN2A acts as an important tumor suppressor gene and is frequently inactivated in somatic leukemic cells [31], germline variants could also impact CDKN2A function during leukemogenesis through either inducing amino acid alteration or expression decreasing. To systematically investigate the influence of SNPs at CDKN2A locus on ALL risk, we conducted a meta-analysis by pooling the ALL-related GWAS and replication studies. Both rs3731217 and rs3731249 exhibits significant associations with ALL risk in this large-scale sample size analysis with 7922 cases/21503 controls and 6295 cases/ 24181 controls respectively. Individuals carrying risk alleles of these two SNPs have 0.72-fold and 2.26-fold increase in disease susceptibility respectively. What’s more, influence of rs3731217 risk allele diverse among ages and ethnicities, possibly because the ALL-associated causal variants are ethnic-specific, or the causal variants can’t be tagged by rs3731217 due to different LD pattern among diverse ethnicities. Similarly, rs3731249 is tend to be an ethnic specific GWAS variant, because the risk allele of rs3731249 were not detected in Asians (MAF = 0, *N* = 4254), rare in Africans (MAF = 0.42%, *N* = 4879), common in Hispanics (MAF = 1.41%, *N* = 5739) and Caucasians (MAF = 3.52%, *N* = 32549) according to the largest exome database (ExAC Browser, http://exac.broadinstitute.org/) [32], indicating the different value of these SNPs on ALL risk assessment across ethnicities. Lack of association for both *CDKN2A* SNPs in Asian population raises a possibility that these two SNPs are in the same LD region, and can be explained by the same causal variant. However, rs3731249 poorly links to rs3731217 across all ethnicities (*r*^2^ < 0.01) (Supplementary Figure S3), and is associated with ALL susceptibility independently according to conditional analysis, suggesting multiple causal variants located within the CDKN2A locus. Moreover, we combined the recent studies and noticed that wild-type allele of rs3731249 is tend to be lost through either loss of heterozygosity or post-transcriptional inactivation during the leukemogenesis process significantly.

By the end of 2017, ∼2110 GWASs had published, reporting around 18000 genome-wide significant (*P* < 5 × 10^–8^) SNPs associated with >1000 traits (http://www.ebi.ac.uk/gwas/) [33], but only a few SNPs identified were considered to be functional and causal, such as rs116855232, a missense SNP induced NUDT15 deficient for 6-mercaptopurine metabolism [34], while the vast majority of GWAS signals were intronic or intergenic SNPs, and considered to tag the nearby causal variants [35]. Great effort has been taken to search the causal variants through fine mapping, or alternatively by exome-array based GWAS, followed by functional analyses. Missense variants are easier to be determined as causal variants by functional analyses or experiments, such as rs1127354 and rs7270101 in ITPA, which can predict the ribavirin-induced anemia [36], and rs3731249 in this study, which induce loss of function of the tumor suppressor p16^INK4A^. Interestingly, we and another independent group also observed that the risk allele is enriched in the leukemic cells through either loss of heterozygosity in DNA level or allele-specific epigenetic modification [14]. In another hand, however, more and more evidence indicate that causal variants are located in the noncoding regions (e.g. promoter, enhancer etc.), and possibly affect the phenotypes by regulating the expression level of the nearby genes, such as rs1427407 in *BCL11A*, which altered the GATA1 and TAL binding motifs of *BCL11A* enhancer region respectively [37]. The systematic analyses have been done to estimate or screen these expression quantitative trait loci (eQTL), including the recently released big project of Genotype-Tissue Expression (GTEx) [38]. Actually, more and more causal GWAS signals were considered to be eQTLs rather than directly affecting the protein coding. In another hand, a recent report demonstrated that rs3731217 was associated with the usage of *CDKN2A* exon 3 [39], which requires more experimental determination but provides an alternative mechanism for GWAS explanation.

## Supporting information

**Supplementary Table 1:** Pooled genotype infromation of rs3731217 polymorphism.

**Supplementary Table 2:** Pooled allele infromation of rs3731217 polymorphism.

**Supplementary Table 3:** Pooled genotype infromation of rs3731249 polymorphism.

**Supplementary Table 4:** Pooled allele infromation of rs3731249 polymorphism.

**Supplementary Table 5:** Sensitivity analysis of rs3731217 polyorphism and ALL risk

**Supplementary Table 6:** Sensitivity analysis of rs3731249 polyorphism and ALL risk

**Supplementary Table 7:** Single nucleotide polymorphism in moderate linkage disequilibrium (r2 < 0.4) with rs3731217 in Caucasians

**Supplementary Table 8:** Hardy Weinberg Equilibrium test for rs3731217 polymorphism

**Supplementary Table 9:** Hardy Weinberg Equilibrium test for rs3731249 polymorphism

**Supplementary Table 10:** Newcastle–Ottawa Quality Assessments scale

**Supplementary Table 11:** Newcastle–Ottawa Quality Assessments of rs3731217 polymorphism

**Supplementary Table 12:** Newcastle–Ottawa Quality Assessments of rs3731249 polymorphism

## Abbreviations

ALL: acute lymphoblastic leukemia
eQTL: expression quantitative trait loci
GWAS: genome-wide association study
LD: linkage disequilibrium
SNP: single nucleotide polymorphism
