# Supplementary material for: Association of the independent polymorphisms in CDKN2A with susceptibility of acute lymphoblastic leukemia
Source: Biosci Rep. 2018 Jun 27;38(3):BSR20180331. doi: 10.1042/BSR20180331 (PMC6019384; doi:10.1042/BSR20180331)
Supplement: Supplementary file 1 [file bsr20180331_Supp1.pdf]

Supplementary Table 1 Pooled genotype infromation of rs3731217 polymorphism.

Author	Country	Year	Ethnicity	Study design	Size		Case			Control		
					Case	Control	TT	TC	CC	TT	TC	CC
Jayaram Vijayakrishnan	UK	2010	Asian	Replication	190	182	148	40	2	138	43	1
Orsi L	France	2012	Caucasian	GWAS	441	1984	338	96	7	1452	486	46
Thomas Burmeister	Berlin	2014	Caucasian	Replication	322	1516	260	60	2	1092	379	32
Agata Pastorczak	Poland	2011	Caucasian	Replication	398	731	304	76	7	551	148	16
Jayaram Vijayakrishnan	UK	2015	Caucasian	GWAS	824	5200	659	149	16	3778	1311	111
			Caucasian	GWAS	834	2024	685	140	9	1523	468	33
Eric A. Hungate et al	USA	2016	Caucasian	Replication	1406	1399	1166	224	16	1052	313	19
			African-American	Replication	203	1363	172	31	0	1044	187	12
			Hispanic	Replication	391	1008	344	46	1	810	157	11
Madara Kreile et al	Latvia	2016	Caucasian	Replication	76	121	64	12	0	95	26	0
Hanene Gharbi et al	Tunis	2016	Afrian	Replication	58	150	13	31	14	21	78	51
Boshra Al-absi et al	Yemen	2017	Asian	Replication	136	153	104	29	3	111	36	6

GWAS, Genome Wide Association study

Supplementary Table 2 Pooled allele infromation of rs3731217 polymorphism.

Author	Country	Year	Ethnicity	Study design	Size		Case			Control		
					Case	Control	T allele	C allele	Total	T allele	C allele	Total
Amy L Sherborne	UK	2010	Caucasian	GWAS	504	1438	899	109	1008	2470	406	2876
Jayaram Vijayakrishnan	UK	2010	Asian	Replication	190	182	336	44	380	319	45	364
Orsi L	France	2012	Caucasian	GWAS	441	1984	772	110	882	3390	578	3968
Thomas Burmeister	Berlin	2014	Caucasian	Replication	322	1516	580	64	644	2563	443	3006
Pauline Peyrouze	France	2012	Caucasian	Replication	150	180	267	33	300	311	49	360
Agata Pastorczak	Poland	2011	Caucasian	Replication	398	731	684	90	774	1250	180	1430
Kyle M. Walsh	USA	2015	Hispanic	Replication	321	454	603	39	642	819	89	908
			Caucasian	Replication	980	2624	1746	214	1960	4471	777	5248
			African-American and Hispanic	Replication	163	201	284	36	320	349	53	402
Jayaram Vijayakrishnan	UK	2015	Caucasian	GWAS	824	5200	1467	181	1648	8867	1533	10400
				GWAS	834	2024	1510	158	1668	3514	534	4048
Anand P. Chokkalingam	USA	2013	Hispanic	Replication	279	406	519	35	554	739	73	812
			Caucasian	Replication	202	369	365	39	404	635	103	738
Eric A. Hungate	USA	2016	Caucasian	Replication	1406	1399	2556	256	2812	2417	351	2768

et al			African-American	Replication	203	1363	375	31	406	2275	211	2486
			Hispanic	Replication	391	1008	734	48	782	1777	179	1956
Madara Kreile et al	Latvia	2016	Caucasian	Replication	76	121	140	12	152	216	26	242
Hanene Gharbi et al	Tunis	2016	Afrian	Replication	58	150	57	59	116	120	180	300
Boshra Al-absi et al	Yemen	2017	Asian	Replication	136	153	237	35	272	258	48	306

GWAS, Genome Wide Association Study

Supplementary Table 3 Pooled genotype infromation of rs3731249 polymorphism.

Author	Country	Year	Ethnicity	Study design	Size		Case			Control		
					Case	Control	CC	CT	TT	CC	CT	TT
Heng Xu et al	USA	2015	Caucasian	GWAS	1773	10441	1541	224	8	9815	619	7
				Replication	409	1599	357	45	7	1467	130	2
Jasmine Healy et al	Canada	2007	French-Canadian	Replication	240	277	195	30	2	256	18	1
Jayaram Vijayakrishnan	UK	2015	Caucasian	GWAS	835	2024	722	101	12	1916	106	2
			Caucasian	GWAS	823	5198	750	69	4	4933	261	4
			Caucasian	Replication	519	1016	472	45	2	974	41	1
Angela Gutierrez-Camino	Spain	2017	Caucasian	Replication	171	234	142	28	1	217	16	1

GWAS, Genome Wide Association Study

Supplementary Table 4 Pooled allele infromation of rs3731249 polymorphism.

Author	Country	Year	Ethnicity	Study design	Size		Case			Control		
					Case	Control	C allele	T allele	Total	C allele	T allele	Total
Heng Xu et al	USA	2015	Caucasian	GWAS	1773	10441	3306	240	3546	20249	633	20882
			Caucasian	Replication	409	1599	759	59	818	3064	134	3198
Jasmine Healy et al	Canada	2007	French-Canadian	Replication	240	277	420	34	454	530	20	550
Jayaram Vijayakrishnan	UK	2015	Caucasian	GWAS	823	5198	1569	77	1646	10127	269	10396
			Caucasian	GWAS	835	2024	1545	125	1670	3938	110	4048
			Caucasian	Replication	519	1016	989	49	1038	1989	43	2032
Kyle M. Walsh	USA	2015	Hispanic	Replication	321	454	609	33	642	889	19	908
			Caucasian	Replication	980	2624	1860	100	1960	5119	129	5248
			African-American and Hispanic	Replication	163	201	306	14	320	398	4	402
Angela Gutierrez-Camino	Spain	2017	Caucasian	Replication	171	234	312	30	342	450	18	468

GWAS, Genome Wide Association Study

Supplementary Table 5 Sensitivity analysis of rs3731217 polyorphism and ALL risk

Omitted Studies				OR	95% CI	P value	I^2
Study	Year	Ethnicity	Study design				
Jayaram Vijayakrishnan et al	2010	Asian	Replication	0.72	0.68 - 0.77	p < 0.00001	0%
Orsi L et al	2012	Caucasian	GWAS	0.71	0.67 - 0.76	p < 0.00001	0%
Thomas Burmeister et al	2014	Caucasian	Replication	0.73	0.68 - 0.78	p < 0.00001	0%
Pauline Peyrouze et al	2012	Caucasian	Replication	0.72	0.68 - 0.77	p < 0.00001	0%
Agata Pastorczak et al	2011	Caucasian	Replication	0.71	0.67 - 0.76	p < 0.00001	0%
Kyle M. Walsh et al	2015	Hispanic	Replication	0.73	0.68 - 0.78	p < 0.00001	0%
		Caucasian	Replication	0.73	0.68 - 0.78	p < 0.00001	0%
		African-American and Hispanic	Replication	0.72	0.68 - 0.77	p < 0.00001	0%
Jayaram Vijayakrishnan et al	2015	Caucasian	GWAS	0.73	0.68 - 0.78	p < 0.00001	0%
			GWAS	0.73	0.68 - 0.78	p < 0.00001	0%

Anand P. Chokkalingam et al	2013	Hispanic	Replication	0.73	0.68 - 0.77	p < 0.00001	0%
		Caucasian	Replication	0.73	0.68 - 0.77	p < 0.00001	0%
Eric A. Hungate et al	2016	Caucasian	Replication	0.73	0.68 - 0.78	p < 0.00001	0%
		African-American	Replication	0.72	0.68 - 0.77	p < 0.00001	0%
		Hispanic	Replication	0.73	0.68 - 0.78	p < 0.00001	0%
Madara Kreile et al	2016	Caucasian	Replication	0.72	0.68 - 0.77	p < 0.00001	0%
Hanene Gharbi et al	2016	Afrian	Replication	0.72	0.68 - 0.77	p < 0.00001	0%
Boshra Al-absi et al	2017	Asian	Replication	0.72	0.68 -0.77	p < 0.00001	0%

GWAS, Genome Wide Association Study; OR, Odds Ratio; CI, Confidence Interval

Supplementary Table 6 Sensitivity analysis of rs3731249 polyorphism and ALL risk

Omitted Studies				OR	95% CI	P value	I^2
Study	Year	Ethinicity	Study design				
Heng Xu et al	2015	European	GWAS	2.23	1.98-2.51	p<0.00001	18%
		European	Replication	2.31	2.10-2.55	p<0.00001	0%
Jasmine Healy et al	2007	European	Replication	2.26	2.06-2.49	p<0.00001	20%
Jayaram Vijayakrishnan et al	2015	European	GWAS	2.33	2.10-2.58	p<0.00001	0%
		European	GWAS	2.17	1.96-2.40	p<0.00001	0%
		European	Replication	2.26	2.05-2.49	p<0.00001	20%
Kyle M. Walsh et al	2015	Hispanic	Replication	2.25	2.05-2.48	p<0.00001	19%
		European	Replication	2.28	2.06-2.52	p<0.00001	19%
		African-American and Hispanic	Replication	2.24	2.04-2.47	p<0.00001	5%
Angela Gutierrez-Camino et al	2017	European	Replication	2.25	2.05-2.48	p<0.00001	20%

GWAS, Genome Wide Association Study; OR, Odds Ratio; CI, Confidence Interval

Supplementary Table 7 Single nucleotide polymorphism in moderate linkage disequilibrium (r2 > 0.4) with rs3731217 in Caucasians

Chr	Pos[a]	LD (r^2)	LD (D')	Variant	Ref	Alt	AFR freq	AMR freq	ASN freq	EUR freq	Motifs changed	GENCODE genes
9	21956078	0.41	-0.86	rs2027938	A	G	0.7	0.85	0.76	0.75	HNF1,Ncx	RP11-145E5.5
9	21956492	0.7	0.85	rs2027939	G	A	0.04	0.09	0	0.15	BDP1,GR,LUN-1	RP11-145E5.5
9	21965232	0.81	0.93	rs2188127	C	G	0.05	0.09	0	0.15	BCL,VDR	RP11-145E5.5
9	21970427	0.82	0.91	rs2518719	A	G	0.13	0.1	0	0.16	NRSF	CDKN2A
9	21980941	0.83	0.92	rs13297747	G	C	0.1	0.1	0.01	0.15	EBF,Pax-5	CDKN2A
9	21983914	1	1	rs3731222	T	C	0.06	0.09	0	0.16	8 altered motifs	CDKN2A
9	21986847	0.44	-1	rs3731211	T	A	0.7	0.82	0.75	0.7	HNF1	CDKN2A
9	21987584	1	1	rs3731204	T	C	0.1	0.1	0	0.16	9 altered motifs	CDKN2A
9	21989477	1	1	rs3731198	T	C	0.04	0.09	0	0.16	Hic1,Klf4,p300	CDKN2A
9	21990457	0.44	-1	rs7036656	C	T	0.72	0.83	0.75	0.7	DMRT5,DMRT7,RFX5	CDKN2A
9	21993964	0.98	1	rs2811711	T	C	0.14	0.1	0	0.16	GCNF,RXRA,VDR	CDKN2A
9	22010412	0.54	0.93	rs495490	A	G	0	0.06	0	0.1	5 altered motifs	RP11-145E5.5
9	22011477	0.54	0.93	rs575427	A	G	0	0.06	0	0.1	NF-kappaB,Pou3f1,p300	RP11-145E5.5

9	22024965	0.54	0.93	rs647188	T	G	0	0.06	0	0.1	Arid5b,Pbx-1,p53	RP11-145E5.5
9	22033389	0.53	0.92	rs1759417	C	T	0	0.06	0	0.1	CEBPD,HMG-IY	CDKN2B-AS1
9	22037071	0.52	0.91	rs35975148	T	A	0	0.06	0	0.1	Pou5f1	CDKN2B-AS1
9	22039426	0.51	0.91	rs12376000	C	T	0	0.06	0	0.1		CDKN2B-AS1

Chr, Chromosome; Pos, Position; LD, Linkage Disequilibrium; Ref, Reference allele; Alt, Alteration allele; AFR, Africans; AMR, Americans; ASN, Asians; EUR, Europeans; GENCODE, Encyclopedia of DNA Elements ,[a] Chromosomal locations are based on hg19.

Supplementary Table 8 Hardy Weinberg Equilibrium test for rs3731217 polymorphism

Author	Country	Year	Ethnicity	Study Design	Size		Case			Control			HWE (Control)	
					Case	Control	TT	TC	CC	TT	TC	CC	Chi-Square	P
Jayaram Vijayakrishnan	UK	2010	Asian	Replication	190	182	148	40	2	138	43	1	1.49	0.22
Orsi L	France	2012	Caucasian	GWAS	441	1984	338	96	7	1452	486	46	0.5	0.49
Thomas Burmeister	Berlin	2014	Caucasian	Replication	322	1516	260	60	2	1092	379	32	0.02	0.9
Agata Pastorczak	Poland	2011	Caucasian	Replication	398	731	304	76	7	551	148	16	2.5	0.11
Jayaram Vijayakrishnan	UK	2015	Caucasian	GWAS	824	5200	659	149	16	3778	1311	111	0.05	0.83
			Caucasian	GWAS	834	2024	685	140	9	1523	468	33	0.19	0.67
Eric A. Hungate et al	USA	2016	Caucasian	Replication	1406	1399	1166	224	16	1052	313	19	0.62	0.43
			African-American	Replication	203	1363	172	31	0	1044	187	12	1.24	0.27
			Hispanic	Replication	391	1008	344	46	1	810	157	11	1.17	0.28
Madara Kreile et al	Latvia	2016	Caucasian	Replication	76	121	64	12	0	95	26	0	1.75	0.19
Hanene Gharbi et al	Tunis	2016	Afrian	Replication	58	150	13	31	14	21	78	51	1.04	0.31
Boshra Al-absi et al	2017	Yemen	Asian	Replication	136	153	104	29	3	111	36	6	1.87	0.17

GWAS, Genome Wide Association Study

Supplementary Table 9 Hardy Weinberg Equilibrium test for rs3731249 polymorphism

Author	Country	Year	Ethnicity	Study design	Size		Case			Control			HWE (Control)	
					Case	Control	CC	CT	TT	CC	CT	TT	Chi-Square	P
Xu Heng et al	USA	2015	Caucasian	GWAS	1773	10441	1541	224	8	9815	619	7	0.75	0.39
				Replication	409	1599	357	45	7	1467	130	2	0.25	0.62
Jasmine Healy et al	Canada	2007	Caucasian	Replication	240	277	195	30	2	256	18	1	1.2	0.27
Jayaram Vijayakrishnan	UK	2015	Caucasian	GWAS	835	2024	722	101	12	1916	106	2	0.18	0.67
			Caucasian	GWAS	823	5198	750	69	4	4933	261	4	0.08	0.77
			Caucasian	Replication	519	1016	472	45	2	974	41	1	0.68	0.41
Angela Gutierrez-Camino et al	2017	Spain	Caucasian	Replication	171	234	142	28	1	217	16	1	1.336	0.248

GWAS, Genome Wide Association Study

Supplementary Table 10 Newcastle–Ottawa Quality Assessments scale

Criteria	Score
**1. The inclusion and exdusion criteria of patients with unexplained recurrent spontaneous abortion**	
Adequate criteria would indude experienced clinical and laboratory examinations	2
Inadequate criteria would indude the patients' history review as the only evidence	1
Not described	0
**2. Source of controls**	
Community controls without history of disease	2
Hospital controls	1
Not described	0
**3. Comparability of cases and controls on the basis of the design or analysis**	
Study controls for select the most important factor	2
Patient medical record	1
Not described	0
**4.Sample size**	
≥200	2
100-199	1
<100	0
**5.Quality control of genotyping methods**	
Repetition of partial/total tested samples with a different method	1
Repetition of partial/total tested samples with the same method	0.5
Not described	0

**6.Hardy-Weinberg equilibrium**	
Hardy-Weinberg equilibrium in control subjects	1
Hardy-Weinberg disequilibrium in control subjects	0
Total	

Supplementary Table 11 Newcastle–Ottawa Quality Assessments of rs3731217 polymorphism

Studies				Quality score
Amy L Sherborne et al	2010	Caucasian	GWAS	8
Jayaram Vijayakrishnan et al	2010	Asian	Replication	7
Orsi L et al	2012	Caucasian	GWAS	10
Thomas Burmeister et al	2014	Caucasian	Replication	8
Pauline Peyrouze et al	2012	Caucasian	Replication	8
Agata Pastorczak et al	2011	Caucasian	Replication	9.5
Kyle M. Walsh et al	2015	Hispanic	Replication	9.5
		Caucasian	Replication	9.5
		African-American and Hispanic	Replication	9.5
Jayaram Vijayakrishnan et al	2015	Caucasian	GWAS	9
		Caucasian	GWAS	9
Anand P. Chokkalingam et al	2013	Hispanic	Replication	9
		Caucasian	Replication	9
Eric A. Hungate et al	2016	Caucasian	Replication	9
		African-American	Replication	9
		Hispanic	Replication	9
Madara Kreile et al	2016	Caucasian	Replication	8
Hanene Gharbi et al	2016	Afrian	Replication	9
Boshra Al-absi et al	2017	Asian	Replication	9

Supplementary Table 12 Newcastle–Ottawa Quality Assessments of rs3731249 polymorphism

Studies				Quality score
Heng Xu et al	2015	Caucasian	GWAS	8
		Caucasian	Replication	8
Jasmine Healy et al	2007	Caucasian	Replication	9
Jayaram Vijayakrishnan et al	2015	Caucasian	GWAS	9
		Caucasian	GWAS	9
		Caucasian	Replication	8
Kyle M. Walsh et al	2015	Hispanic	Replication	9.5
		Caucasian	Replication	9.5
		African-American and Hispanic	Replication	9.5
Angela Gutierrez-Camino et al	2017	Caucasian	Replications	9.5
